# Electrophysiological Evidence of Early Auditory Dysfunction in Personal Listening Device Users: Insights from ABR with Ipsilateral Masking

**DOI:** 10.3390/diagnostics15212672

**Published:** 2025-10-23

**Authors:** A. P. Divya, Praveen Prakash, Sreeraj Konadath, Reesha Oovattil Hussain, Vijaya Kumar Narne, Sunil Kumar Ravi

**Affiliations:** 1 Department of Audiology, All India Institute of Speech and Hearing, Mysore 570006, India; dapdivya@gmail.com; 2 JSS Institute of Speech and Hearing, Dharwad 580007, India; praveenprakashp1849@gmail.com; 3 Department of Medical Rehabilitation Sciences, College of Applied Medical Sciences, King Khalid University, Abha 61481, Saudi Arabia; rehussain@kku.edu.sa (R.O.H.); vnarne@kku.edu.sa (V.K.N.); sravi@kku.edu.sa (S.K.R.)

**Keywords:** personal listening devices, hidden hearing loss, extended high-frequency audiometry, auditory brainstem response, ipsilateral masking, noise-induced auditory dysfunction

## Abstract

**Background:** Recreational noise exposure from personal listening devices (PLDs) may lead to hidden hearing loss (HHL), affecting auditory nerve function despite normal pure-tone audiometry (PTA) and otoacoustic emissions (OAE). Subclinical auditory damage at the synaptic level often goes undetected by conventional assessments, emphasizing the need for more sensitive measures. Recorded click ABR in the presence of various levels of ipsilateral maskers for the better identification of auditory damage at the synaptic level. These results could help to develop a better objective diagnostic tool that can detect hidden hearing loss. **Objective:** To examine the effects of PLD usage on extended high-frequency audiometric thresholds and on click-evoked auditory brainstem responses (ABR) with and without ipsilateral masking in individuals with normal hearing. **Materials and Methods:** Thirty-five young adults aged 18–35 years (18 PLD users, 17 controls) with clinically normal hearing were recruited. Extended high-frequency audiometry (EHFA) was conducted from 9 to 16 kHz. Click-evoked ABRs were recorded at 80 dB nHL under unmasked and ipsilateral broadband noise-masked conditions at 50, 60, and 70 dB SPL. ABR analyses included absolute and relative amplitude (V/I) and latencies of waves I, III, and V. **Results:** PLD users demonstrated significantly elevated extended high-frequency thresholds compared to controls. ABR analyses revealed reduced Wave I amplitudes across stimulus conditions in PLD users, while Wave V amplitudes were largely preserved, resulting in consistently higher V/I amplitude ratios under masked conditions. No group differences were observed for Wave III amplitudes or absolute/interpeak latencies, except for a modest prolongation of I–III latency at one masker level in PLD users. **Conclusions:** Conventional audiological tests may not detect early auditory damage; however, extended high-frequency audiometry and ABR with ipsilateral masking demonstrate greater sensitivity in identifying noise-induced functional changes within the auditory brainstem pathways.

## 1. Introduction

Noise exposure is a pervasive threat to auditory health, stemming from a variety of sources, including automobiles, events, construction sites, concerts, industrial processes, entertainment venues, and personal listening devices [1]. Many national and international organizations have issued recommendations over the past few decades to safeguard human health against the negative impacts of exposure to environmental noise [2] (Occupational Safety and Health Administration (OSHA), National Institute for Occupational Safety and Health (NIOSH), World Health Organization (WHO), etc.). Although the audiological impacts of occupational noise exposures are well-documented, there is limited understanding of how other frequently encountered sources of noise contribute to an individual’s total noise exposure. This is a matter of serious concern as excessive noise has been shown to have negative health impacts, regardless of the source. A large proportion of noise exposure in young individuals occurs as a part of social and recreational activities involving loud music [3]. Despite being voluntary, this exposure can lead to hearing loss and other health issues over time. Listening to music at high volume levels can subject listeners to potentially harmful noise for extended periods, particularly when using portable music players and other recreational noise sources. Although young individuals are generally aware of the harmful effects of high noise levels, they may not fully recognize that personal listening devices, such as mobile phones and portable MP3 players, often produce sound at potentially dangerous levels [3].

Literature reports have revealed that our auditory system is vulnerable to considerable damage resulting from noise exposure, even before the individual experiences the common symptoms of auditory damage, such as ear pain, tinnitus, or difficulty understanding speech in noisy environments [3]. Lower Middle Ear Muscle Reflex (MEMR) amplitudes measured using wideband probes have been found to be associated with higher levels of lifetime noise exposure [4]. Individuals with noise exposure tend to exhibit reduced response levels and a narrower range in both click-evoked otoacoustic emissions (CEOAEs) and distortion-product otoacoustic emissions (DPOAEs) [5]. Although awareness of recreational noise risks has improved, several studies continue to report an insufficient understanding and lack of preventive behavior among young adults and personal listening device users [6]. The classification of the observed structural alterations remains uncertain, as it is often challenging to disentangle the effects of noise exposure from those of other contributing factors that can affect auditory integrity. Recent animal studies suggest that even before noticeable hearing loss occurs, a more subtle and common process may cause lasting damage to the synaptic connections between inner hair cells and certain cochlear nerve fibers [7]. Glutamate receptor expression temporarily decreases, possibly as a protective measure, during short-term synapse reshaping. Low-SR fibers on the modiolar side appear most vulnerable to noise damage [8]. Acoustic trauma disrupts auditory dendrites, leading to overstimulation of glutamatergic inner hair cells (IHCs). These variables encompass both dependent variables that assess the effects of exposure and independent variables that characterize the exposure conditions [9]. Excitotoxicity is a complicated process of excessive glutamate release that occurs when noise overstimulation causes glutamate receptors on Spiral Ganglion Neuron (SGN) postsynaptic membranes to become overactivated, which underlies degenerative neuronal cell death [9].

Determining how noise affects the inner ear in NIHL is a challenging task. Prior to auditory abnormalities manifesting on an audiogram, OAEs demonstrate cumulative damage to the inner ear. Thus, in a noise-hazardous setting, decreased OAEs are considered predictive of future hearing loss [10]. Hair cell function is evaluated by Electrocochleography (ECoG) using the SP/AP parameter. While the majority of ECoG investigations focus on identifying endolymphatic hydrops, some have also reported cochlear cell injury from SNHL and NIHL [11]. Even at frequencies where hearing thresholds are within normal limits, a reduction in acoustic reflex amplitudes was evidently observed in several studies conducted on individuals with NIHL [12]. Numerous studies have also demonstrated that prolonged noise exposure impairs attention control and central auditory processing, and causes concomitant behavioral abnormalities [13,14].

There is various evidence to suggest that recreational NIHL, especially through Personal Listening Devices (PLDs), should be taken into account as a public health risk. According to WHO estimates, harmful listening patterns when using PLDs could put 1.1 billion young people under 35 years old at risk of hearing damage [15]. Over the past decade, there has been an exponential increase in the number of PLDs that can play music loudly, particularly in developing nations such as India [16]. With PLD’s greater battery life, portability, and greater storage capacity, it is anticipated that the prevalence of noise exposure from PLDs has increased alongside technical improvements [17]. This has allowed PLD users to listen to a wider variety of higher-quality content for longer periods of time, in conjunction to rising mobile data rates and the availability of online media content. Due to the widespread use of PLDs and the associated noise exposure, a substantial portion of the community is regularly exposed to noise, even at low levels, which may increase their risk of exceeding recommended exposure limits when other noise sources are considered [18]. The estimated SPLs produced by PLDs are found to vary from 79 to 125 dBA due to several variables, including the earphone seal against the ear, player output voltage, earphone sensitivity, and recorded music levels and type of PLD (For example, earphone seal, as using intra-concha “earbud” earphones increases the risk of excessive sound levels), the listening habits of the user, the kind of earphone, including fit, the music genre, the impact of background noise levels, etc. Due to their proximity to the tympanic membrane and potential for higher sound levels than those produced by conventional home or commercial sound systems, personal stereo systems that use earphones are a subject of concern [17].

Several studies have focused on the effects of PLD usage in the auditory system. Although the precise influence of PLD users’ listening habits on hearing thresholds is unknown, they are significantly affected when listening to music with background noise exceeding 65 dB compared to non-PLD users [19]. In a study by Sulaiman et al. [20] on PLD users who were listening at 50% of their gadget’s maximum volume for one hour each day, the audiograms did not show typical indicators of NIHL. Their audiometric thresholds at the majority of the standard test frequencies (0.25–8 kHz) were similar to those of the controls. However, in contrast, the PLD users’ mean hearing thresholds were noticeably greater than those of the controls for many of the extended high frequencies (9–16 kHz). Moreover, PLD users’ TEOAE and DPOAE amplitudes were lower than those of controls. These findings suggested that the PLD user group were in the early stage of high-level sound-induced hearing impairment. Bal and Derinsu [21] examined ECoG and ABR measures in personal listening device users with normal hearing sensitivity and no otologic or neurological disorders. Their findings indicated that users exposed to higher listening levels showed lower neural response amplitudes, reflecting early cochlear or neural changes consistent with hidden hearing loss.

Individuals with normal audiometric thresholds may still experience suprathreshold auditory deficits, a condition commonly referred to as hidden hearing loss (HHL). Emerging evidence suggests that HHL may stem from cochlear synaptopathy—a loss of synaptic connections between inner hair cells and auditory nerve fibers, particularly those with low spontaneous firing rates. However, findings across studies remain inconsistent, despite several reports describing altered audiological measures in individuals who are likely to exhibit early neural changes associated with cochlear synaptopathy. Fulbright et al. [22] investigated relationships between noise exposure history, ABR wave I amplitude, and suprathreshold performance in individuals with normal audiometric thresholds, reporting no significant associations among these measures. In contrast, Bramhall et al. [23] observed a modest but statistically significant correlation between lower ABR wave I amplitudes and poorer speech-in-noise recognition in a broader hearing cohort, particularly in ears with slightly elevated pure-tone thresholds. Together, these findings highlight the variability and complexity in relating ABR amplitude to suprathreshold speech perception, likely influenced by differences in subject selection, audiometric status, noise-exposure assessment, and electrophysiologic protocols. Additional studies also suggest that subjective measures may be less sensitive in detecting early noise-induced changes [24], while objective ABR measures show variability across individuals, which can impact the sensitivity and generalizability of these tests [25].

According to Suresh and Krishnan [26], there is little consensus across different behavioral and audiological test measures; hence, the gold standard for diagnosing cochlear synaptopathy remains the histological analysis of a harvested temporal bone by autopsy. The authors examined the results of click-evoked ABR with and without ipsilateral masking noise, conventional Pure Tone Audiometry, High-Frequency Audiometry, and Distortion Product Oto-Acoustic Emissions (DPOAEs) in 28 normal-hearing adults who had participated in a marching band for five or more years (the high-risk group) and 28 age-matched controls (the low-risk group). The groups’ DPOAE amplitudes and audiometric thresholds did not differ significantly from one another. In the click-evoked ABR with a simultaneous ipsilateral masking condition, the high-risk group’s wave I amplitude decreased more slowly than that of the low-risk group, with an increase in background noise level, suggesting a reduced masking effect. In contrast, the high-risk group exhibited a smaller wave I amplitude and an enhanced V/I amplitude ratio at moderate and high stimulus levels.

Since low-spontaneous rate (low-SR) fibers are more vulnerable to glutamate excitotoxicity [27], manipulating the auditory brainstem response (ABR) with suprathreshold ipsilateral broadband noise (BBN) at varying click-to-BBN ratios provides a more sensitive means of probing auditory neural integrity. This approach helps dissociate the contributions of low-SR fibers from those of high- and mid-SR fibers, thereby offering greater resolution in identifying early synaptic dysfunction. By increasing the physiological load on the auditory pathway, masked ABR paradigms are particularly well-suited to reveal subtle neural deficits that often remain undetected in conventional ABR recordings obtained in quiet conditions. Although several studies have examined the effects of PLD use on cochlear and behavioral measures in individuals with normal audiograms, the application of masked ABR to this population remains scarce. Given its heightened sensitivity to neural damage at the synaptic level, ipsilateral masked ABR represents a promising objective biomarker for hidden hearing loss. In light of this gap, the present study aimed to investigate the impact of PLD use on auditory function by combining extended high-frequency audiometry with click-evoked ABR under both unmasked and ipsilateral noise-masked conditions. Together, these approaches offer a more comprehensive and sensitive assessment of early auditory changes that may precede conventional audiometric deficits.

## 2. Method

### 2.1. Participants

Thirty-five right-handed individuals (35 ears) with clinically normal hearing participated in the study. The study followed a comparative case–control design. The overall age range of the participants varied from 18 to 35 years (mean 24.6 years, S. D = 3.7 years). All participants were in good general health, with no history of otological, neurological, or systemic disorders that could affect auditory function. The participants were divided into two groups: personal listening device (PLD) users, who formed the experimental group, and individuals with normal hearing and no significant use of listening devices, who served as controls. The experimental group consisted of 18 individuals (9 males and 9 females) and the control group consisted of 17 individuals (8 males and 9 females). The age ranges of the participants in the experimental and control groups were 18 to 35 years (mean 25.2 ± 2.52 years) and 18 to 33 years (mean 24.3 ± 3.0 years), respectively. Apart from the individuals in the experimental group, who were exposed to loud sound levels via listening devices, none of the participants were subject to occupational or any other consistent source of loud noise, nor did they have any risk factors for developing hearing loss.

### 2.2. Ethical Considerations

Before participants were recruited for the study, the objectives, test procedures, average duration, and possible implications of the study were explained, and written informed consent was obtained from each participant. All procedures were non-invasive, were conducted in accordance with the ethical standards of the Institutional Ethics Committee (Approval No.: JSSISH/EC/P22/2023) and adhered to the principles outlined in the Declaration of Helsinki.

### 2.3. Participant Selection

All the participants underwent a detailed audiological evaluation before they were subjected to the test procedures of the study. A detailed case history probing to the present and past events focusing on possible and potential symptoms for any otological/auditory damage was carried out. Any cases of systemic disease and/or related medication, habitual alcohol consumption, smoking, or any neurological conditions were marked as exclusionary criteria.

An otoscopic evaluation and immittance audiometry (incorporating a 226 Hz probe tone) were performed to ensure normal outer and middle ear structure and function. Any individuals with tympanometric findings other than ‘A’ or ‘As’ and with absent or elevated acoustic reflex thresholds for 500, 1000, 2000, and 4000 Hz stimuli were excluded from the study. The immittance evaluation was conducted using a GSI TympStar (Grason-Stadler, Eden Prairie, MN, USA) immittance audiometer [28].

The pure tone audiometry was performed at both octaves as well as mid-octave frequencies ranging from 250 Hz to 8000 Hz using a GSI Audiostar Pro (Grason-Stadler, Eden Prairie, MN, USA) dual-channel clinical audiometer [28]. The stimulus presentation for air conduction and bone conduction was carried out using Telephonics TDH-50P (Telephonics Corporation, Farmingdale, NY, USA) supra-aural headphones and Radio ear B-71 bone vibrator, respectively. The pure tone average (PTA) was calculated for frequencies of 500, 1000, 2000, and 4000 Hz, and all participants had a PTA of 15 dB HL or lower, with all hearing thresholds at individual test frequencies within 20 dB HL. The bone conduction thresholds were measured at each test frequency to confirm that participants’ hearing thresholds were within the normal range and to rule out any undiagnosed conductive hearing loss, ensuring that all thresholds reflected true cochlear sensitivity. All participants had a speech identification score of 95 percent or higher. The distortion product otoacoustic emission (DPOAE) screening was performed using an ILOV 6 OAE instrument to ensure a clinically healthy cochlear function. The DPOAE measurements were obtained using two tones, f1 and f2 (primaries), in the range of 500–4000 Hz, with a f2/f1 ratio of 1.22, and intensities of 65 dB SPL and 55 dB SPL (L1 and L2), respectively. A 3 dB signal-to-noise ratio (SNR) criterion was used as a screening threshold for determining DPOAE pass/fail. This threshold is sufficient to reliably detect cochlear outer hair cell function while minimizing false negatives due to minor background noise fluctuations and is widely accepted in both clinical and research settings. A higher SNR threshold (e.g., 6 dB) was not applied, as our study targeted participants with normal hearing but significant recreational sound exposure, who might exhibit subtle cochlear changes that could be excluded by stricter criteria. All audiological test procedures were conducted in an acoustically treated, double-room setup with noise levels within permissible limits [29].

### 2.4. Grouping of Participants

A structured interview was carried out to categorize participants into experimental and control groups. All participants, both in the experimental and control groups, were recruited from the general population and not from clinical patients presenting with hearing complaints. Recruitment followed the same structured interview protocol to classify participants based on PLD usage, ensuring consistency between groups. The interview emphasized parameters of earphone usage such as duration per day/week, volume levels, the preferred genre of music, the purpose of usage (professional musician/instrumentalist or personal recreational usage), daily life situations, and the background noise levels where they used the device, (while traveling in a bus or train, while driving open/closed vehicles), other listening devices, and frequency of attending social/recreational activities involving high noise levels. Based on the interview, participants who had no significant PLD usage (<1 h per week and volume levels approximating less than 80 dB SPL or no usage at all) were enrolled in the control group. Individuals with considerable use of PLD for more than one hour per day and habitual listening (for at least one year) with volume levels greater than 80 dB SPL were enrolled in the experimental group. Although the volume levels were further measured quantitatively, as an informal and initial approach, the statement and questions emphasizing the intensity of the volume levels were addressed in the interview in terms of a volume progression bar that appeared on the screen while changing the volume levels. To verify their reports on habitual listening levels, the participants in the experimental group were asked to record their listening levels for a one-week period.

### 2.5. Real Ear Measurements of SPL Generated by PLDs

The ‘Aurical Free Fit’ Real ear measurement system was used to measure the output sound pressure levels generated by the PLDs. The equipment was calibrated, and the subjects were positioned in a soundproof room at a pre-determined distance. A probe microphone was introduced into the subject’s ear canal. An otoscopic evaluation was performed to verify the position of the probe microphone within a few millimeters of the tympanic membrane. An on-ear measurement of the ambient background levels was taken as a baseline to guarantee precise microphone placement and operation. The participant’s PLD was positioned over the ear’s probe microphone, which locked it in place, and the corresponding earphone was inserted. Each participant was instructed to play a song from their preferred genre at their usual volume level. Sound pressure levels generated by the PLD (wired/wireless and headphones) inside the ear canal at the level of the tympanic membrane were obtained at from the Long-Term Average Speech Spectrum (LTASS) by analyzing during the ‘Live’ mode of the instrument. Based on real-ear measurements (REMs), participants’ self-reported listening levels were objectively verified. Participants were included in the experimental group if the REMs confirmed that their sound levels exceeded 80 dB SPL, and in the control group if the REMs confirmed levels below 80 dB SPL, in accordance with the inclusion criteria established during the structured interview.

### 2.6. High-Frequency Audiometry and Auditory Brainstem Response

Following routine audiological evaluations, which included otoscopy, immittance, pure tone evaluations, and OAE screening, the participants underwent extended high-frequency (EHF) audiometry and auditory brainstem response evaluations.

### 2.7. Extended High-Frequency Audiometry

The EHF audiometry was conducted at 9 kHz, 10 kHz, 11.2 kHz, 12.5 kHz, 14 kHz, and 16 kHz using a Sennheiser HDA-200 earphone (Wedemark, Germany), employing the Hughson–Westlake method to obtain audiometric thresholds [30].

### 2.8. Auditory Brainstem Response

The auditory brainstem responses were obtained using click stimulus (100 microseconds) using EEG data acquisition equipment and stimulus generator (Intelligent Hearing Systems, SmartEP software, version 5.55.00). The stimulus was presented at 80 dB nHL to obtain the unmasked ABR and at the same level in the presence of a wideband ipsilateral masker at three different masking levels: 50, 60, and 70 dB SPL. These masker levels (50–70 dB SPL) were selected because they were comfortable for participants while being sufficient to assess the impact of masking on ABR amplitude and latency. An intensity level of 80 dB nHL was chosen for this experiment for two major reasons: (a) compared to lower stimulus levels, waveforms recorded at 80 dB made it easy to identify and plot the peaks in response waveforms, and (b) compared to 90 dB, 80 dB nHL was softer in loudness, and as the testing involved sweep runs at multiple masker levels, softer intensity was chose to avoid any possible discomfort that the participant/s could experience.

Data Acquisition: To minimize myogenic artifacts, the participants were asked to relax and avoid unnecessary body movements while lying comfortably in an electro-acoustically shielded booth. For the entire duration of the recording sessions, participants were advised to ignore the test stimulus and remain calm by maintaining their eyes closed and, if possible, sleeping. To improve test outcomes, the skin impedance was reduced to less than 3 kΩ, with no inter-electrode impedance exceeding 1 kΩ, by using skin prep gel (Weaver Nuprep Skin Preparation Gel). The electrodes were positioned with the help of skin conduction paste (Weaver Ten20 Conductive EEG paste) and were securely attached with surgical plaster. A single-channel recording was carried out, which involved three separate electrodes: non-inverting, inverting, and ground. These electrodes were placed on the high forehead, close to the hairline, on the test ear mastoid, and on the non-test ear mastoid. The stimuli were presented to the participant’s right ear through an ER-3 insert earphone (Etymotic Research, ER-3). The clicks were presented at an 11.1/s rate using rarefaction polarity. The order of the stimulus presentation was randomized for every participant to avoid any possible stimulus order-related fatigue in neural responses. The EEG waves were band-pass filtered between 100 and 3000 Hz and amplified by 100,000 times. Each ABR waveform obtained was the result of an average of 1500 digitalized stimulus sweeps over a 12-ms analysis epoch. The artifact rejection value of ±31 μV was automatically applied to exclude stimulus-related epochs tainted by muscle or movement artifacts from the averaging process.

ABR analysis: Two complete recordings of 1500 sweep waveforms were obtained. Visual overlay cursors were utilized on a computer screen to show the peak-to-trough amplitudes. Separate ABR identification, latency, and amplitude observations of waves I, III, and V were obtained for both unmasked and masked conditions by two audiologists with over 10 years of experience in recording, analyzing, and interpreting auditory-evoked potentials, with a primary focus on the ABR. This followed a blinded procedure where they were instructed to mark the peaks on the waveforms present in each individual page provided in the IHS software user interface generator (Intelligent Hearing Systems, SmartEP software, version 5.55.00), where the two recordings obtained for each stimulus condition were moved randomly to different pages. No information regarding the participant or group characteristics was provided to the examining audiologists. Any disagreements amongst two audiologists were settled by a third professional who had an equivalent or higher experience level. After successfully marking the peaks with no or resolved discrepancies, the absolute amplitude and latency, as well as interpeak latencies, were noted, and the V/I amplitude ratio was calculated. This was carried out for all the masker conditions, and the obtained data parameters were subjected to between-group and groupwise analyses.

### 2.9. Statistical Analyses

The obtained data were analyzed using the Statistical Package for the Social Sciences (SPSS) software, version 27.0. The Shapiro–Wilk test indicated normal distribution of EHF thresholds and all ABR parameters (*p* > 0.05). Between-group comparisons for EHF thresholds and ABR amplitude measures were performed using independent samples *t*-tests. Repeated-measures (RM) ANOVA was conducted for within-subject comparisons of ABR amplitudes and interpeak latencies across masker conditions. post hoc pairwise comparisons following RM-ANOVA were adjusted using the Bonferroni correction to control for type I error.

## 3. Results

### 3.1. Extended High-Frequency Audiometry Findings

**Figure 1:**
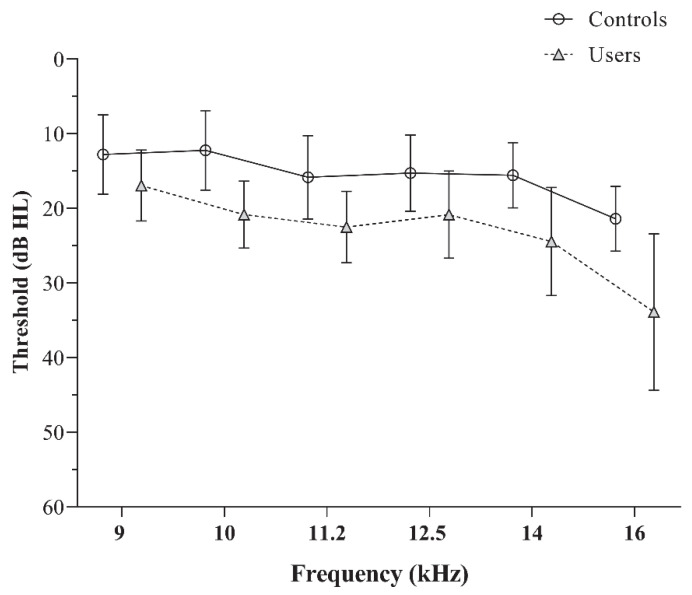
The mean and standard deviation of the extended high-frequency audiometric thresholds obtained by the controls and PLD users.

The EHF audiometry revealed significantly elevated thresholds for PLD users compared to controls, with consistent observations across all test frequencies (*p* < 0.05). The mean and standard deviation of pure tone thresholds for the two groups are depicted in Figure 1. The independent *t*-test values used to compare the EHF thresholds are presented in Table 1.

### 3.2. Absolute Amplitude of ABR Peaks

The between-group comparison of the absolute amplitudes of peaks I, III, and V was carried out using independent *t*-tests with a Bonferroni-adjusted α value of 0.0125. The findings revealed a significantly reduced peak I amplitude in PLD users across all four stimulus conditions (unmasked and the three masker levels), compared to non-users (*p* < 0.01).

For peak III, no significant differences were observed between the groups for any of the stimulus conditions. Similarly, for peak V, there were no differences between the two groups under any stimulus conditions (*p* > 0.05), except for the 70 dB masker condition, in which the PLD users group exhibited a significantly higher peak V amplitude than the control group (*p* < 0.01). The findings of the independent *t*-tests comparing the absolute amplitudes obtained under different stimulus conditions between the groups are presented in Table 2.

The effect of masker levels on the absolute amplitudes of peaks I, III, and V were analyzed using RM-ANOVA, conducted separately for each group. For peak I, both controls and PLD users showed a reduction in mean amplitude with increasing masker intensity compared to the unmasked condition [F(controls) (3, 51) = 10.59, *p* < 0.001, ηp^2^ = 0.38; F(users) (3, 48) = 19.18, *p* < 0.001, ηp^2^ = 0.55].

Pairwise comparisons using a Bonferroni-adjusted α value of 0.008 revealed a statistically significant reduction in amplitude at the 70 dB masker level relative to the unmasked, 50 dB, and 60 dB levels. This effect was consistently observed in both groups (*p* < 0.008).

For peak III, although a main effect of masker level was observed for both controls [F(3, 51) = 4.31, *p* < 0.01, ηp^2^ = 0.21] and PLD users [F(3, 48) = 5.86, *p* < 0.01, ηp^2^ = 0.27], pairwise comparisons revealed no statistically significant differences across any of the stimulus conditions for either group (*p* > 0.08).

**Figure 2:**
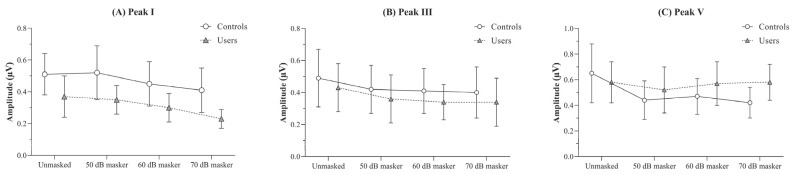
Figure showing the mean and standard deviation of absolute amplitude (in µV) obtained under the unmasked, and 50, 60, and 70 dB SPL masker levels for (**A**) peak I, (**B**) peak III, and (**C**) peak V by the PLD users and the controls.

Similarly, to peak I, controls showed a significantly reduced peak V amplitude with increasing masker intensity compared to the unmasked condition [F(controls) (3, 51) = 15.48, *p* < 0.001, ηp^2^ = 0.48]. In contrast, PLD users exhibited no significant differences in peak V amplitude across the stimulus conditions [F(users) (3, 48) = 0.15, *p* > 0.05, ηp^2^ = 0.01]. The absolute amplitudes obtained by the two groups under the unmasked and different masker intensity conditions are illustrated in Figure 2.

### 3.3. V/I Amplitude Ratio

**Figure 3:**
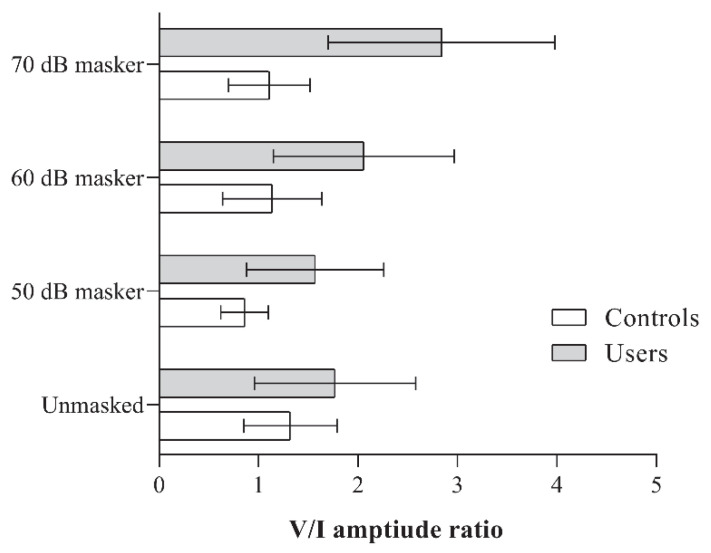
Figure showing the V/I amplitude ratio obtained by the two groups (PLD users and controls) for the unmasked, and 50, 60, and 70 dB SPL masker levels.

There were no significant differences in the V/I amplitude ratio between the two groups under the unmasked condition (*p* > 0.05). However, under all three masking conditions, PLD users consistently exhibited a significantly higher V/I amplitude ratio (*p* < 0.01). The mean amplitude ratios obtained by the groups under different stimulus conditions, along with the results of the independent *t*-tests, are presented in Figure 3 and Table 3, respectively.

When comparing within-group differences across the different stimulus conditions, controls showed a reduced V/I amplitude ratio at the 50 dB masker level compared to the unmasked condition. A significant main effect was observed for the non-user group [F(3, 51) = 5.06, *p* < 0.001, ηp^2^ = 0.23]. No significant differences were found between the other stimulus condition pairs within this group.

In contrast, PLD users demonstrated a significantly higher V/I amplitude ratio at the 70 dB masker level compared to the other stimulus conditions (*p* < 0.008), with a significant main effect observed [F(3, 48) = 16.49, *p* < 0.001, ηp^2^ = 0.51]. Although the mean amplitude ratios at the 50- and 60 dB masker levels were higher than the unmasked condition (refer to Figure 3), they did not reach the adjusted significance threshold of 0.008.

### 3.4. Absolute Latency of ABR Peaks

There were no significant differences in the absolute latencies of peaks I, III, and V between the two groups across any of the four stimulus conditions (*p* > 0.05).

The results of the RM-ANOVA conducted to examine the effect of masker intensity on absolute latencies within each group revealed a trend of increasing mean latency with increasing masker intensity. This effect was statistically significant for both the controls [F(3, 51) = 4.20, *p* < 0.05, η^2^_p_ = 0.19] and the users [F(3, 48) = 7.02, *p* < 0.001, η^2^_p_ = 0.30]. However, despite the presence of a significant main effect, pairwise comparisons between stimulus conditions did not yield any differences that reached the adjusted significance level (*p* < 0.008) in either group.

For peak III, both controls and users showed significant latency prolongations across masker levels [F(3, 51) = 30.74, *p* < 0.001, η^2^_p_ = 0.64 for controls; F(3, 48) = 28.63, *p* < 0.001, η^2^_p_ = 0.64 for users].

**Figure 4:**
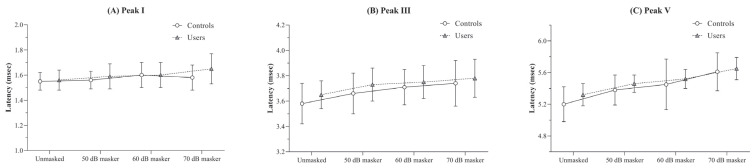
Figure showing the mean and standard deviation of absolute latency (in ms) obtained under the unmasked, and 50, 60, and 70 dB SPL masker levels for (**A**) peak I, (**B**) peak III, and (**C**) peak V by the PLD users and the controls.

A similar trend was observed for peak V, where both groups exhibited prolonged latencies with increasing masker levels [F(3, 51) = 18.87, *p* < 0.001, η^2^_p_ = 0.53 for controls; F(3, 48) = 43.82, *p* < 0.001, η^2^_p_ = 0.73 for users]. The mean and standard deviation of absolute latencies for peaks I, III, and V across different stimulus conditions for both groups are illustrated in Figure 4.

### 3.5. ABR Interpeak Latencies

**Figure 5:**
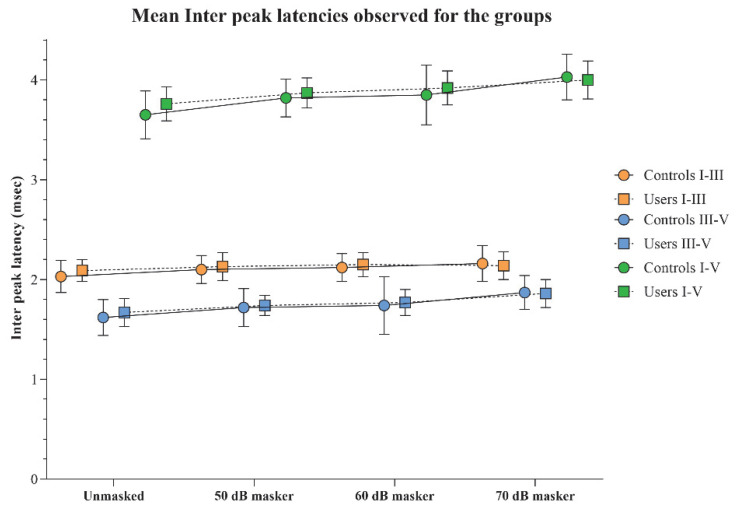
The mean and standard deviation of interpeak latency (in ms) for I–III, III–V, and I–V obtained under the unmasked, and 50, 60, and 70 dB SPL masker levels by the PLD users and the controls.

The interpeak latencies I–III, III–V, and I–V obtained for the two groups were compared using independent *t*-tests, which revealed no significant differences between the groups for any of the stimulus conditions (*p* > 0.05). This trend was consistent across all interpeak latency measures. The mean and standard deviation of the interpeak latencies for each stimulus condition in both groups are presented in Figure 5.

Within-group comparisons of interpeak latencies across different stimulus conditions, analyzed using RM-ANOVA, revealed significant prolongations in I–III, III–V, and I–V latencies with increasing masker intensities (*p* < 0.008), along with considerable effect sizes.

A notable exception was observed for the I–III interpeak latency in the PLD users’ group. Although a main effect indicating latency prolongation with increasing masker intensity was present, a statistically significant difference was observed only between the 60 dB masker and the unmasked condition (*p* < 0.008). Additionally, the overall effect size for the I–III latency in the PLD users was comparatively smaller (η^2^_p_ = 0.16) than for other latency measures, and the same was observed in the control group. The RM-ANOVA results are shown in Table 4.

## 4. Discussion

The current study investigated the impact of personal listening device (PLD) use on auditory brainstem responses (ABRs) in individuals with normal audiometric thresholds at routine clinical frequencies (250–8000 Hz), both with and without an ipsilateral masker, as well as extended high-frequency (EHF) assessment. All participants reported no difficulty hearing in quiet or noisy environments, despite subtle elevations in EHF thresholds. Participants were enrolled based on a structured interview regarding their daily duration, preferred levels, and overall use of PLDs, with Real Ear Measurements used to verify reported listening levels.

Although all participants exhibited normal pure-tone thresholds, individuals with significant PLD usage showed elevated EHF thresholds compared to controls across all frequencies except 8 kHz. This aligns with prior findings [3,31], where PLD users demonstrated higher thresholds in the 10–20 kHz range despite normal conventional audiometry. These results emphasize that EHF audiometry can serve as an early indicator of cochlear changes before conventional thresholds are affected. The initial signs of auditory damage from noise exposure often affect the outer hair cells at the base of the cochlea, which are responsible for encoding higher-frequency sounds. These frequencies are not routinely examined in conventional audiometry, as the standard range primarily assesses speech-relevant frequencies. However, EHF testing is particularly valuable in populations with noise exposure, as it can detect early cochlear changes before conventional thresholds are affected [31].

In the current study, most participants had been using PLDs for fewer than five years on average. The absence of clinically significant noise-induced hearing loss (NIHL) in standard pure-tone audiometry (PTA) results may be attributable to this relatively short exposure period.

Turning to electrophysiological findings, PLD users exhibited significantly reduced Wave I amplitudes across all stimulus conditions. Notably, these differences were magnified under higher ipsilateral masker levels, indicating that masked ABR paradigms enhance sensitivity to subtle neural dysfunction. While reductions in Wave I amplitude have been reported previously in other noise-exposed populations [26,32,33], the present study extends these findings specifically to habitual PLD users, highlighting the utility of masked ABR in this increasingly relevant population. The progressive reduction in Wave I amplitude with increasing masker intensity supports the view that low-spontaneous-rate auditory nerve fibers, which are most vulnerable to synaptopathic damage, are preferentially stressed under these conditions [26,34]. This demonstrates that masked ABR can detect early synaptic changes even in individuals with normal audiograms, offering complementary information to EHF audiometry. While EHF thresholds provide early cochlear-level indicators of damage, masked ABR reveals functional deficits at the auditory nerve level, which may precede the onset of threshold shifts.

The observed profile of reduced Wave I amplitude with preserved Wave V amplitude in PLD users closely mirrors the characteristics of cochlear synaptopathy [26,32,33]. This suggests that masked ABR may serve as a non-invasive marker of hidden hearing loss, capturing early neural changes not evident in conventional audiometry or OAEs. Human temporal bone studies have shown that age-related reductions in Wave I amplitude correlate with loss of spiral ganglion cells and synaptic ribbons, supporting the link between reduced suprathreshold ABR and synaptic loss [26,35].

Wave III amplitude showed minimal differences between groups, consistent with previous animal and human studies indicating central compensatory gain mechanisms in response to peripheral auditory nerve deficits [36]. In the present study, Wave V amplitude was largely unaffected by group differences, except at the 70 dB masker condition, where PLD users showed significantly higher peak V amplitudes than controls. Consistent with this, multiple animal studies on synaptopathy have also reported that ABR waves III and V amplitudes remain preserved despite reductions in auditory nerve input, highlighting potential central compensatory mechanisms [37,38,39,40]. The preserved or enhanced central response, alongside reduced peripheral input, reflects homeostatic central gain mechanisms aimed at maintaining auditory function in the face of reduced cochlear input [37,38,39,40,41]. The increase in V/I amplitude ratios under masked conditions further supports the use of this metric as a sensitive electrophysiological marker of subclinical auditory dysfunction.

Multiple studies investigating tinnitus patients with normal hearing thresholds, many of whom have a history of noise exposure, have consistently reported increased Wave V amplitudes in their auditory brainstem responses (ABR) [42,43]. This suggests that central auditory circuits increase gain to compensate for reduced cochlear input, which may help preserve perception but can also contribute to the perception of tinnitus [43]. Exposure to noise can impair hair cell synapses and disrupt auditory nerve input, with central mechanisms attempting to restore activity, as reflected by higher Wave V amplitudes even when audiograms remain normal [44,45].

Latency analyses revealed no significant group differences across most conditions, though interpeak latencies generally increased with masker intensity. A notable exception was the I–III interpeak latency in PLD users between unmasked and 60 dB SPL masker conditions, which did not reach significance. This may reflect a ceiling effect in neural disruption, where compensatory mechanisms in the lower brainstem limit further latency prolongation. Similar latency shifts due to ipsilateral masking have been reported in previous studies, indicating disrupted neural synchrony under noisy conditions [46].

Collectively, these findings highlight the complementary utility of masked ABR and EHF audiometry. While EHF thresholds detect early cochlear-level damage, masked ABR reveals subclinical neural deficits, particularly in low-SR fibers that are vulnerable to noise exposure. This dual assessment approach enhances the early identification of at-risk individuals, such as habitual PLD users, and provides a rationale for incorporating masked ABR in clinical evaluations where conventional audiometry may appear normal. Early detection through these methods could inform preventive strategies and longitudinal monitoring, particularly in populations exposed to high-level sound from recreational or occupational sources.

## 5. Conclusions

The present study aimed to identify early changes in the auditory pathway associated with the use of personal listening devices. Findings revealed elevated extended high-frequency thresholds and reduced Wave I amplitudes in PLD users, while Wave V responses were preserved, indicating possible central gain compensation. These results suggest that conventional audiological tests may overlook subclinical auditory deficits. In contrast, extended high-frequency audiometry and ABR with ipsilateral masking provide greater sensitivity in detecting noise-induced functional alterations in the auditory brainstem. Importantly, the masked ABR paradigm demonstrated enhanced ability to reveal subtle neural dysfunction, underscoring its potential as an objective clinical tool for the early detection of hidden hearing loss in individuals with normal audiograms.

## Figures and Tables

**Table 1:** Independent *t*-test results comparing EHF thresholds between groups across frequencies. Mean differences (in dB HL), t-values, degrees of freedom (df), *p*-values, and Cohen’s *d* are reported.

Frequency (Hz)	9 k	10 k	11.2 k	12.5 k	14 k	16 k
Mean Difference	−4.17	−8.61	−6.67	−5.56	−8.89	−12.50
t	−2.41	−5.10	−3.73	−2.95	−4.33	−4.54
df	34.00	34.00	34.00	34.00	34.00	34.00
*p*	0.02	<0.01	<0.01	<0.01	<0.01	<0.01
Cohen’s *d*	−0.80	−1.70	−1.24	−0.98	−1.44	−1.51

**Table 2:** Independent *t*-test results comparing the absolute amplitude of peaks I, III, and V obtained by the two groups under different stimulus conditions. Mean differences (in dB HL), t-values, degrees of freedom (df), *p*-values, and Cohen’s *d* are reported. Note: Bolded mean values indicate statistically significant differences at *p* < 0.01.

Peak I				
Conditions	Unmasked	50 dB SPL	60 dB SPL	70 dB SPL
Mean Difference	**0.14**	**0.17**	**0.15**	**0.18**
t	3.19	3.60	3.72	4.87
df	33.00	33.00	33.00	33.00
*p*	<0.01	<0.01	<0.01	<0.01
Cohen’s *d*	1.08	1.22	1.26	1.65
**Peak III**				
Mean Difference	0.06	0.06	0.06	0.06
t	1.05	1.25	1.44	1.11
df	33.00	33.00	33.00	33.00
*p*	0.30	0.22	0.16	0.27
Cohen’s *d*	0.35	0.42	0.49	0.38
**Peak V**				
Mean Difference	0.08	−0.09	−0.09	**−0.16**
t	1.14	−1.51	−1.70	−3.69
df	33.00	33.00	33.00	33.00
*p*	0.26	0.14	0.10	<0.01
Cohen’s *d*	0.39	−0.51	−0.57	−1.25

**Table 3:** Table shows the test statistic values of the independent *t*-test comparing the V/I amplitude ratio obtained by the non-users and PLD users under different stimulus conditions. Note: Bolded mean values indicate statistically significant differences at *p* < 0.01.

Conditions	Unmasked	50 dB SPL	60 dB SPL	70 dB SPL
Mean Difference	−0.45	**−0.72**	**−0.92**	**−1.72**
t	−1.97	−4.05	−3.60	−5.87
df	33.00	33.00	33.00	33.00
*p*	0.06	<0.001	<0.01	<0.001
Cohen’s *d*	−0.67	−1.37	−1.22	−1.98

**Table 4:** Presents the results of RM-ANOVA conducted to examine the effect of different stimulus conditions on interpeak latencies (I–III, III–V, and I–V) within each group (Controls and PLD Users). Note: The symbol ‘*’ next to the F-values indicates statistical significance—*p* < 0.001—and F value without ‘*’ indicates significance: *p* < 0.05.

		F	η^2^_p_
Controls I–V d*f* (3, 51)	I–III	14.24 *	0.46
	III–V	8.14 *	0.32
	I–V	19.13 *	0.53
Users I–III d*f* (3, 48)	I–III	3.12	0.16
	III–V	15.1 *	0.49
	I–V	16.25 *	0.5

## Data Availability

The data presented in this study are available on request from the corresponding author due to being a part of an ongoing study.

## References

[1] Le Clercq C.M.P., Goedegebure A., Jaddoe V.W.V., Raat H., De Jong R.J.B., Van Der Schroeff M.P. (2018). Association between portable music player use and hearing loss among children of school age in the Netherlands. JAMA Otolaryngol. Head Neck Surg..

[2] van Kempen E., Casas M., Pershagen G., Foraster M. (2018). WHO environmental noise guidelines for the European region: A systematic review on environmental noise and cardiovascular and metabolic effects: A summary. Int. J. Environ. Res. Public Health.

[3] Peng J.H., Tao Z.Z., Huang Z.W. (2007). Risk of damage to hearing from personal listening devices in young adults. J. Otolaryngol..

[4] Shehorn J., Strelcyk O., Zahorik P. (2020). Associations between speech recognition at high levels, the middle ear muscle reflex and noise exposure in individuals with normal audiograms. Hear. Res..

[5] Attias J., Horovitz G., El-Hatib N., Nageris B. (2001). Detection and Clinical Diagnosis of Noise-Induced Hearing Loss by Otoacoustic Emissions. Noise Health.

[6] Levey S., Levey T., Fligor B.J. (2011). Noise exposure estimates of urban MP3 player users. J. Speech Lang. Hear. Res..

[7] Liberman M.C., Kujawa S.G. (2017). Cochlear synaptopathy in acquired sensorineural hearing loss: Manifestations and mechanisms. Hear. Res..

[8] Liberman L.D., Liberman M.C. (2015). Dynamics of cochlear synaptopathy after acoustic overexposure. JARO J. Assoc. Res. Otolaryngol..

[9] Lai T.W., Zhang S., Wang Y.T. (2014). Excitotoxicity and stroke: Identifying novel targets for neuroprotection. Prog. Neurobiol..

[10] Lapsley Miller J.A., Marshall L., Heller L.M., Hughes L.M. (2006). Low-level otoacoustic emissions may predict susceptibility to noise-induced hearing loss. J. Acoust. Soc. Am..

[11] Wong A.C.Y., Ryan A.F. (2015). Mechanisms of sensorineural cell damage, death and survival in the cochlea. Front. Aging Neurosci..

[12] Houghton J.M., Greville K.A., Keith W.J. (1988). Acoustic reflex amplitude and noise-induced hearing loss. Int. J. Audiol..

[13] Chiovenda P., Pasqualetti P., Zappasodi F., Ercolani M., Milazzo D., Tomei G., Capozzella A., Tomei F., Rossini P.M., Tecchio F. (2007). Environmental noise-exposed workers: Event-related potentials, neuropsychological and mood assessment. Int. J. Psychophysiol..

[14] Kujala T., Shtyrov Y., Winkler I., Saher M., Tervaniemi M., Sallinen M., Teder-Sälejärvi W., Alho K., Reinikainen K., Näätänen R. (2004). Long-term exposure to noise impairs cortical sound processing and attention control. Psychophysiology.

[15] World Health Organization (WHO) (2015). World Report on Hearing.

[16] Basu S., Garg S., Singh M., Kohli C. (2019). Knowledge and practices related to the use of personal audio devices and associated health risks among medical students in Delhi. J. Educ. Health Promot..

[17] Keith S.E., Michaud D.S., Chiu V. (2008). Evaluating the maximum playback sound levels from portable digital audio players. J. Acoust. Soc. Am..

[18] Gilliver M., Nguyen J., Beach E.F., Barr C. (2017). Personal Listening Devices in Australia: Patterns of Use and Levels of Risk. Semin. Hear..

[19] Jiang W., Zhao F., Guderley N., Manchaiah V. (2016). Daily music exposure dose and hearing problems using personal listening devices in adolescents and young adults: A systematic review. Int. J. Audiol..

[20] Sulaiman A.H., Husain R., Seluakumaran K. (2014). Evaluation of early hearing damage in personal listening device users using extended high-frequency audiometry and otoacoustic emissions. Eur. Arch. Otorhinolaryngol..

[21] Bal N., Derinsu U. (2021). The possibility of cochlear synaptopathy in young people using a personal listening device. Auris Nasus Larynx.

[22] Fulbright A.N.C., Le Prell C.G., Griffiths S.K., Lobarinas E. (2017). Effects of Recreational Noise on Threshold and Suprathreshold Measures of Auditory Function. Semin. Hear..

[23] Bramhall N., Ong B., Ko J., Parker M. (2015). Speech perception ability in noise is correlated with auditory brainstem response wave I amplitude. J. Am. Acad. Audiol..

[24] Prendergast G., Millman R.E., Guest H., Munro K.J., Kluk K., Dewey R.S., Hall D.A., Heinz M.G., Plack C.J. (2017). Effects of noise exposure on young adults with normal audiograms II: Behavioral measures. Hear. Res..

[25] Stamper G.C., Johnson T.A. (2015). Letter to the Editor: Examination of Potential Sex Influences in Stamper. Auditory Function in Normal-Hearing, Noise-Exposed Human Ears, Ear Hear, 36, 172–184. Ear Hear..

[26] Suresh C.H., Krishnan A. (2021). Search for Electrophysiological Indices of Hidden Hearing Loss in Humans: Click Auditory Brainstem Response Across Sound Levels and in Background Noise. Ear Hear..

[27] Furman A.C., Kujawa S.G., Liberman M.C. (2013). Noise-induced cochlear neuropathy is selective for fibers with low spontaneous rates. J. Neurophysiol..

[28] Grason-Stadler, Inc GSI-61 Clinical Audiometer. https://acmerevival.com/wp-content/uploads/2021/01/GSI-61-Brochure1-1.pdf.

[29] American National Standards Institute (ANSI) (2010). ANSI S3.6–2010: Specification for Audiometers.

[30] Carhart R., Jerger J.F. (1959). Preferred Method for Clinical Determination of Pure-Tone Thresholds. J. Speech Hear. Disord..

[31] Škerková M., Kovalová M., Mrázková E. (2021). High-frequency audiometry for early detection of hearing loss: A narrative review. Int. J. Environ. Res. Public Health.

[32] Bramhall N.F. (2021). Use of the auditory brainstem response for assessment of cochlear synaptopathy in humans. J. Acoust. Soc. Am..

[33] Barbee C.M., James J.A., Park J.H., Smith E.M., Johnson C.E., Clifton S., Danhauer J.L. (2018). Effectiveness of Auditory Measures for Detecting Hidden Hearing Loss and/or Cochlear Synaptopathy: A Systematic review. Semin. Hear..

[34] Kujawa S.G., Liberman M.C. (2009). Adding insult to injury: Cochlear nerve degeneration after “temporary” noise-induced hearing loss. J. Neurosci..

[35] Kujawa S.G., Liberman M.C. (2006). Acceleration of age-related hearing loss by early noise exposure: Evidence of a misspent youth. J. Neurosci..

[36] Wang Q., Yang L., Qian M., Hong Y., Wang X., Huang Z., Wu H. (2021). Acute Recreational Noise-Induced Cochlear Synaptic Dysfunction in Humans with Normal Hearing: A Prospective Cohort Study. Front. Neurosci..

[37] Caspary D.M., Ling L., Turner J.G., Hughes L.F. (2008). Inhibitory Neurotransmission, Plasticity and Aging in the Mammalian Central Auditory System. J. Exp. Biol..

[38] Schrode K.M., Muniak M.A., Kim Y.H., Lauer A.M. (2018). Central compensation in auditory brainstem after damaging noise exposure. eNeuro.

[39] Melcher J.R., Kiang N.Y. (1996). Generators of the brainstem auditory evoked potential in cat III: Identified cell populations. Hear. Res..

[40] Schaette R., McAlpine D. (2011). Tinnitus with a normal audiogram: Physiological evidence for hidden hearing loss and computational model. J. Neurosci..

[41] Hickox A.E., Larsen E., Heinz M.G., Shinobu L., Whitton J.P. (2017). Translational issues in cochlear synaptopathy. Hear. Res..

[42] Chambers A.R., Resnik J., Yuan Y., Whitton J.P., Edge A.S., Liberman M.C., Polley D.B. (2016). Central Gain Restores Auditory Processing Following Near-Complete Cochlear Denervation. Neuron.

[43] Hickox A.E., Liberman M.C. (2014). Is noise-induced cochlear neuropathy key to the generation of hyperacusis or tinnitus?. J. Neurophysiol..

[44] Caspary D.M., Schatteman T.A., Hughes L.F. (2005). Age-related changes in the inhibitory response properties of dorsal cochlear nucleus output neurons: Role of inhibitory inputs. J. Neurosci..

[45] Salvi R.J., Wang J., Ding D. (2000). Auditory plasticity and hyperactivity following cochlear damage. Hear. Res..

[46] Burkard R., Hecox K. (1983). The effect of broadband noise on the human brainstem auditory evoked response. II. Frequency specificity. J. Acoust. Soc. Am..

